# Effects of Tai Chi on the neuromuscular function of the patients with functional ankle instability: a study protocol for a randomized controlled trial

**DOI:** 10.1186/s13063-022-06046-w

**Published:** 2022-02-02

**Authors:** Huiru Tang, Min Mao, Daniel T. P. Fong, Qipeng Song, Yan Chen, Zhipeng Zhou, Cui Zhang, Jiangna Wang, Xuewen Tian, Wei Sun

**Affiliations:** 1 College of Sports and Health, Shandong Sport University, Jinan, China; 2 School of Nursing and Rehabilitation, Shandong University, Jinan, China; 3 National Centre for Sport and Exercise Medicine, School of Sport, Exercise and Health Sciences, Loughborough University, Loughborough, UK; 4 Lab of Biomechanics, Shandong Institute of Sport Science, Jinan, China

**Keywords:** Tai Chi, Neuromuscular function, Functional ankle instability, Balance, Ankle proprioception

## Abstract

**Background:**

Ankle instability limits physical activities and undermines a person’s quality of life. Tai Chi’s health benefits have been reported in different population groups. However, the effects of Tai Chi on neuromuscular function among young adults with functional ankle instability (FAI) remain unclear. Therefore, we aim to investigate the effect of Tai Chi on young adults with FAI.

**Methods:**

This study will be conducted as a randomized controlled trial with blinded assessors. A total of 104 young adults with FAI will be recruited and randomly assigned to intervention and control groups. The participants in the simplified Tai Chi exercise program (STCEP) group will receive a 12-week Tai Chi training. The participants in the control group will receive a low-intensity exercise program and health education for 12 weeks. The primary and secondary outcomes will be assessed at baseline, 4th, 8th, and 12th weeks. Primary outcome measures will include the Cumberland Ankle Instability Tool (CAIT) score, kinematics/kinetics data, electromyography during single-leg landing tasks, and the modified Star Excursion Balance Test (mSEBT). Secondary outcome measures will include the total time of Dynamic Leap and Balance Test (DLBT), ankle muscle strength, and ankle proprioception.

**Discussion:**

This study will investigate the effects of Tai Chi exercise on the neuromuscular function of patients with FAI, as indicated by ankle joint biomechanics, ankle proprioception, balance, ankle muscle strength, and ankle muscle activation. Results will demonstrate that Tai Chi can be an effective exercise for young adults with FAI.

**Trial registration:**

Chinese Clinical Trial Registry ChiCTR2100044089. Registered on 10 March 2021

## Administrative information

Note: the numbers in curly brackets in this protocol refer to SPIRIT checklist item numbers. The order of the items has been modified to group similar items (see http://www.equator-network.org/reporting-guidelines/spirit-2013-statement-defining-standard-protocol-items-for-clinical-trials/).

**Table:** 

Title {1}	Effects of Tai Chi on the neuromuscular function of the patients with functional ankle instability: a study protocol for a randomized controlled trial
Title {1}	Effects of Tai Chi on the neuromuscular function of the patients with functional ankle instability: a study protocol for a randomized controlled trial
Trial registration {2a and 2b}.	Chinese Clinical Trial Registry ChiCTR2100044089. Registered on 10 March 2021. https://www.chictr.org.cn
Protocol version {3}	The protocol is in version 1.0. Dated Sep 30, 2021.
Funding {4}	This study was supported by Shandong Provincial Natural Science Foundation (ZR2020QC091), China Shandong Key Research and Development Plan (2019GSF108211, 2020CXGC010902), National Natural Science Foundation of China (31700815).
Author details {5a}	Huiru Tang^1^, Min Mao^2^, Daniel T.P. Fong^3^, Qipeng Song^1^, Yan Chen^1^, Zhipeng Zhou^1^, Cui Zhang^4^, Jiangna Wang^1^, Xuewen Tian^1^, Wei Sun^1^* 1. College of Sports and Health, Shandong Sport University, Jinan, China 2. School of nursing and rehabilitation, Shandong University, Jinan, China 3. National Centre for Sport and Exercise Medicine, School of Sport, Exercise and Health Sciences, Loughborough University, Loughborough, UK 4. Lab of Biomechanics, Shandong Institute of Sport Science, Jinan, China
Name and contact information for the trial sponsor {5b}	Not applicable. There is no sponsor for this study.
Role of sponsor {5c}	The funder has no input in the study design, protocol preparation, or future data analysis and interpretation.

## Introduction

### Background and rationale {6a}

Acute ankle sprain is one of the most common musculoskeletal injuries in sports [1]. Approximately 40–75% of ankle sprains may lead to chronic ankle instability (CAI) [2]. Functional ankle instability (FAI) is the most common type of CAI. The symptoms of FAI include giving away or subjective ankle instability, sensorimotor impairments, and a high ankle sprain recurrence rate [3]. Individuals with FAI have a substantially higher risk of ankle abnormal biomechanics, which may lead to ankle osteoarthritis. This condition will adversely affect the daily living activities of patients with FAI [2].

Neuromuscular control at the ankle provides the unconscious activation of dynamic restraints that occur in preparation for and in response to ankle joint motion and loading [4]. Repeated ankle sprains may lead to neuromuscular deficits, which significantly impair the ankle stability [5]. Patients with FAI have deficits on joint position sense on the affected side compared with those of the healthy contralateral side [6]. Caulfield et al. collected the surface electromyography (sEMG) of peroneus longus of patients with FAI during landing tasks. They found that activation in the peroneus longus muscle reduces during the experimental period immediately prior to initial contact compared with that of the healthy control [7]. In addition, Delahunt et al. found that patients with FAI have a reduced dorsiflexion position and a decreased sagittal plane angular velocity during landing compared with those of the healthy control [8]. Therefore, the neuromuscular function of patients with FAI should be improved to prevent and treat ankle sprain injury.

Regular exercise training is essential for the rehabilitation of patients with FAI [9]. One of the traditional Chinese forms of exercise is Tai Chi, which has many characteristics that can help patients with FAI improve their neuromuscular control problems. Tai Chi is characterized by continuous slow movements with unilateral to bilateral shifts of body weight, a large range of motions at the joints, and smooth foot movement in various directions [10]. Slow, steady, and coordinated movements likely improve joint proprioception; they also activate and strengthen deep ankle muscles [11]. Therefore, the primary emphasis on posture control may make Tai Chi a relevant and appropriate alternative exercise for neuromuscular deficits among patients with FAI. Recently, one study has applied Tai Chi to treat FAI. A study has shown that 12-week traditional 24-form Tai Chi training can improve the Cumberland Ankle Instability Tool (CAIT) score and the modified Star Excursion Balance Test (mSEBT) performance [12]. However, they are insufficient to prove that Tai Chi is effective for patients with FAI. The effects of Tai Chi exercise on neuromuscular function, joint biomechanics, and physical functioning in individuals with FAI should be further investigated to understand the clinical effects and relevant mechanism of the treatment.

In this study, a simplified Tai Chi exercise program (STCEP) specifically for patients with FAI will be developed. A randomized controlled trial will be conducted to investigate the clinical efficacy of 12-week STCEP on neuromuscular function. The results of the present study will determine the effectiveness and provide scientific evidence to establish a form of comprehensive sports rehabilitation for patients with FAI.

### Objectives {7}

The primary objective is to assess the effect of STCEP on the neuromuscular function of patients with FAI. We hypothesize that STCEP will be effective for improving the neuromuscular function of patients with FAI.

Eligible participants will be randomized at a 1:1 ratio and enrolled in one of the two groups. Two intervention programs will be included in this study, namely, intrinsic-foot-muscle exercise combined with lower extremity resistance training (IFM), extrinsic-foot-muscle exercise combined lower extremity resistance training (EFM), and lower extremity resistance training program.

### Trial design {8}

**Fig. 1:**
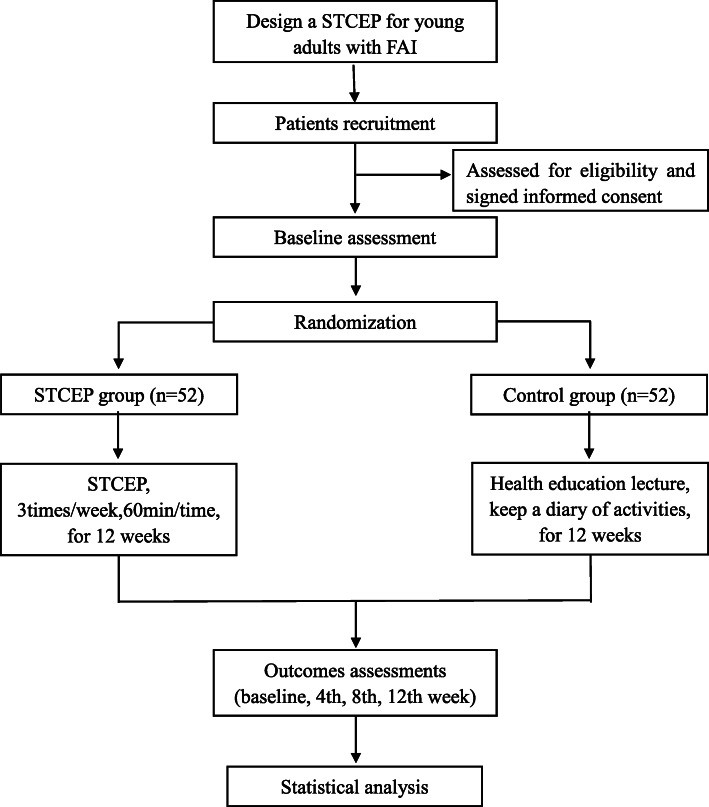
An overview of the study

The effectiveness of STCEP on young adults with FAI will be studied using a randomized, single-blind, and parallel-controlled trial. Eligible participants will be randomized at a 1:1 ratio and enrolled in one of the two groups with allocation concealment and assessor blinding. The study will be carried out over a 12-week intervention (i.e., a simplified Tai Chi exercise program). An overview of the study is displayed in Fig. 1.

## Methods: participants, interventions, and outcomes

### Study setting {9}

The participants will be recruited, assessed, and subjected to intervention at Shandong Sport University, China.

### Eligibility criteria {10}

The inclusion and exclusion criteria are determined using the recommendations of the International Ankle Consortium [13].

#### Inclusion criteria

1. The initial sprain must have occurred at least 12 months prior to study enrollment, associated with inflammatory symptoms (pain and swelling), and created at least one interrupted day of the desired physical activity.
2. They experienced at least two episodes of giving way in the 6 months prior to study enrollment.
3. Their CAIT score must be ≤24.

#### Exclusion criteria

1. Positive anterior drawer test/Talar tilt test
2. Already a Tai Chi practitioner
3. Currently enrolled in an ankle rehabilitation program
4. Other muscular, joint, or neurological conditions that affect the lower limb function
5. Visual, vestibular, and cognitive deficits

### Who will take informed consent? {26a}

After the patients are initially screened in accordance with the inclusion and exclusion criteria, eligible volunteers who agreed to participate will sign a written informed consent before the intervention. The informed consent process will be performed by the main researchers (HT) to explain the purpose, procedures, potential risks, benefits, and confidentiality of the information in detail.

### Additional consent provisions for collection and use of participant data and biological specimens {26b}

On the consent form, participants have the right to withdraw at any time and will be asked if they agree to the use of their data should they choose to withdraw from the trial. The participants will also be asked for permission for the research team to share relevant data with people from the universities taking part in the research or from regulatory authorities where relevant. This trial does not involve collecting biological specimens for storage.

## Interventions

### Explanation for the choice of comparators {6b}

FAI may be associated with the altered muscle activation ratio, impaired proprioception, and neuromuscular control. Several studies have found that Tai Chi, as a traditional Chinese exercise form, can improve ankle proprioception, static balance, lower extremity muscle strength, and neuromuscular reaction [11]. It has been widely used to treat patients with Parkinson’s disease, stroke, and knee osteoarthritis [14–16]. Tai Chi exercise may offer potential benefits to patients with FAI. However, the advantages for these patients who practice Tai Chi exercise as rehabilitation have not been rigorously tested. Therefore, the effects of Tai Chi exercise on neuromuscular function, joint biomechanics, and physical functioning in individuals with FAI should be further investigated to understand the clinical effects and relevant mechanism of the treatment. In this study, the participants in the STCEP group will receive a simplified Tai Chi. The STCEP emphasizes ankle joint movements and aims to promote the neuromuscular control of patients with FAI.

### Intervention description {11a}

#### STCEP group

**Fig. 2:**
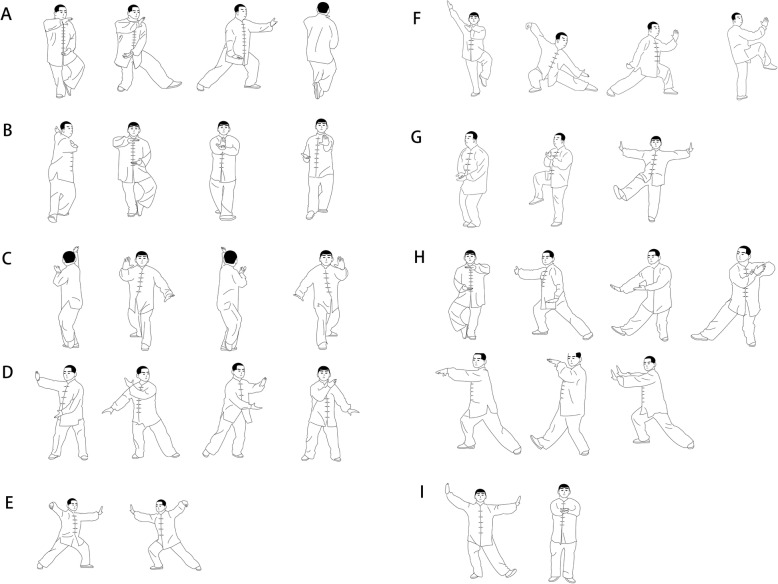
The core program consisting of nine forms of STCEP

This STCEP is derived from the traditional Yang style, and the core program consists of nine forms of STCEP (Fig. 2):

Form A: part the wild horse’s mane (stepping diagonally forward: left and right)

Form B: step back up and whirl arms (stepping backward: left and right)

Form C: brush knee and twist step (stepping forward: left and right)

Form D: cloud hands. Form E: single whip (stepping sideways: left and right)

Form F: pushing down and single on one leg (stepping diagonally forward: left and right)

Form G: heel kick (in situ: left and right)

Form H: grasp the bird’s tail (stepping sideways: left and right)

Form I: cross-hand (stepping sideways: left and right)

The exercise routine emphasizes the practice of ankle joint movements with more repetitions in multiple directions, which may help improve the neuromuscular function of ankle joints in patients with FAI. These movements are usually maintained in a semi-squat and one-leg stance, controlling the displacement of the center of gravity in three anatomical planes. The coordination of all body parts plays a vital role in maintaining precise motor control while keeping balance. Any simple action in the traditional 24-style Tai Chi program that involves fewer foot-ankle movements is removed. Afterward, the rest of the movements are rearranged according to the suggestions of Tai Chi experts. The exercise program will be specially designed to promote the neuromuscular control of patients with FAI.

The participants in the STCEP group will receive 12 weeks of the STCEP training, three sessions per week, and 60 min per session. Each session will include 5 min warm-up exercises, 50 min practice, and 5 min cool-down exercises. Every training session will be led and monitored by a professional Tai Chi instructor, who will carefully guide the exercise and correct inappropriate movements.

#### Control group

The participants in the control group will attend a low-intensity exercise program and receive health education but without similar training benefits in lower extremity weight bearing, strength, or balance [14]. The low-intensity exercise program will consist of whole body stretches, controlling breathing, and relaxation. Health education will cover health-related topics, such as ankle sprain, prevention, and rehabilitation. The weekly schedule will be identical to that of the STCEP group, and each session will include 40 min exercise and 20 min health education. Every training session will be led and monitored by a professional exercise instructor who will carefully guide the exercise and correct inappropriate movements.

### Criteria for discontinuing or modifying allocated interventions {11b}

Study patients will be allowed or asked to withdraw from the study if:

1. Participants decide to withdraw
2. Participants develop serious diseases, such as heart disease or stroke
3. Participants have an adverse reaction related to the STCEP program
4. Participants missed more than three training sessions in the 12-week program

### Strategies to improve adherence to interventions {11c}

The subjects’ attendance in every session will be recorded by the research assistant (who does not participate in follow-up evaluation and data analysis). The detailed exercise content and benefits will be informed to the participants to promote participant retention. The patients will be contacted and encouraged through WeChat three times a week. They will be asked about the factors that hinder their activities and solve them in time. In addition, teaching videos will be provided according to the teaching process to promote their exercise enthusiasm.

### Relevant concomitant care permitted or prohibited during the trial {11d}

During the trial, the participants will be informed to avoid taking part in any other regular rehabilitation training and maintain their previous lifestyle.

### Provisions for post-trial care {30}

Not applicable. There is no anticipated harm and compensation for trial participation.

### Outcomes {12}

**Fig. 3:**
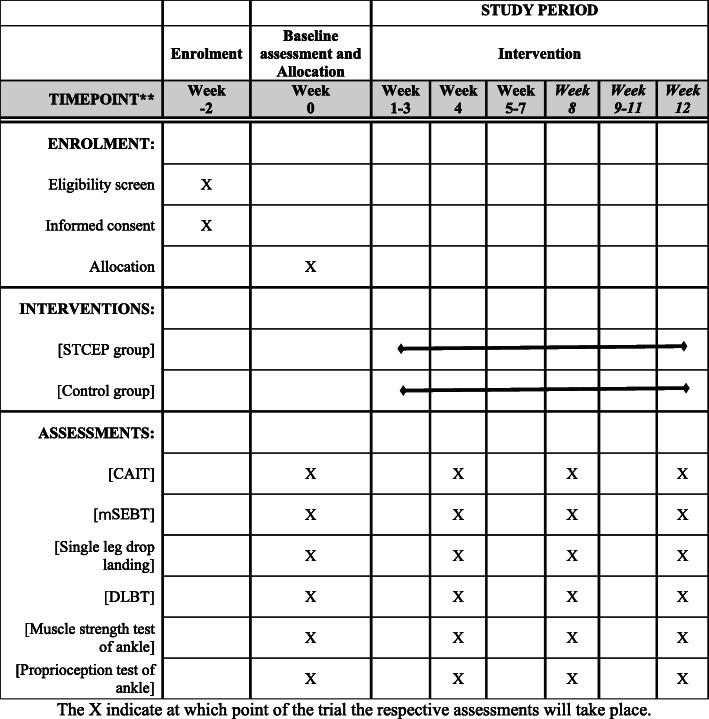
The time schedule of enrolment, visits for participants, intervention, and assessments

Demographic information, primary outcomes, and secondary outcomes will be collected. Demographic information will be collected at baseline (1–2 weeks before randomization) via a questionnaire. Primary and secondary outcomes will be measured at baseline, 4th, 8th, and 12th weeks of training. The schedule of study outcome assessments is outlined in Fig. 3.

#### Primary outcome assessments

1. Cumberland Ankle Instability Tool
  The CAIT is a valid and reliable evaluative instrument used to assess self-rated ankle instability [17]. It consists of eight items for ankle instability in sports/daily activities and one item for ankle pain. The scores from the nine items are added to obtain a final score. The final score ranges from 0 (worst) to 30 (best).
2. Biomechanical analysis during mSEBT
  mSEBT is a test that has been commonly utilized as a modified version of the SEBT to dynamic balance in clinics for the CAI groups [18]. In this test, participants stand on a single leg on the center of a force platform with hands on the waist as the starting position for the test. They maintain a single-leg stance and slowly reach the contralateral leg as far as they could along the anterior, posteromedial, or posterolateral directions. They tap their toes without shifting the body weight to the reaching limb and then return to the starting position while maintaining their single-leg stance balance for about 10 s. They perform three trials on each foot. In this study, the mean distance from the three trials will be used for analysis.
  Kinetic data will be simultaneously collected using two force plates (AMTI) with a sampling frequency of 1000 Hz in combination with the mEMG of the leg muscles for the mSEBT test. Rectangular electrodes with parallel bar electrodes are placed bilaterally over the muscle belly of the rectus femoris, biceps femoris, tibialis anterior, and peroneus longus [19].
3. Biomechanical analysis during the single-leg drop landing
  A 40-cm-high box is placed 5 cm posterior to the force plate [20]. Before the test trial, the subjects are provided three trials to practice the maneuver. The participants are first asked to stand on the box with hands on the waist. The examiner gives the verbal command of “Ready,” and the participants stick out the tested leg and keep balance. As soon as hearing the command of “Start,” the participants jump straight down from the box, land on the tested leg at the center of the force plate, and stabilize as quickly as possible. They keep the single-leg stance balance for at least 5 s. Between trials, a 30-s rest will be provided. A trial will be discarded and repeated if the participants lose balance after landing or use their hands/feet are used to restore balance.
  Lower extremity kinematic data will be obtained using a three-dimensional motion capture system by 12 infrared cameras at 100 Hz (Vicon, Oxford, UK) and in combination with ground reaction force data, which will be measured by two force plates (AMTI) with a sampling frequency of 1000 Hz and in combination with the sEMG of the leg muscles for the landing task. The markers will be placed bilaterally on the first metatarsal head, first and fifth metatarsal phalangeal joints, medial and lateral malleoli, posterior calcaneus, medial and lateral femoral epicondyles, greater trochanters, anterior iliac spine, iliac crest, posterior iliac spine, and the fourth lumbar vertebra.

#### Secondary outcome assessments

1. Muscle strength test of the ankle

The isokinetic strength of the ankle muscles will be measured using an isokinetic dynamometer (IsoMed 2000). An isokinetic test will be conducted at angular velocities of 30°/s [21]. During the ankle plantar and dorsal flexor test, the participants will lay supine on the dynamometer seat with their lower limbs in full extension. The foot will be placed on the foot adapter connected to the head of a dynamometer with an ankle angle at 0° of plantar flexion (neutral position of the talocrural joint). Static gravitational correction will be applied to negate the influence of the gravity-effect torque on the test data. During the ankle invertor and evertor testing, the head of the dynamometer and the test seat are adjusted to 80° and 45°, respectively. The participants will be seated on the dynamometer seat with their shin positioned horizontally to the ground. Three trials will be performed for each direction on each foot. The mean of three trials will be used for the analysis.

## Proprioception test of ankle

Ankle proprioception data will be collected using the Sunny proprioception instrumentation [22]. The participants will sit on a chair with the testing leg placed on the platform during the test. The participants will be asked to wear eye masks and noise-canceling headphones to eliminate any visual and auditory stimuli from the test environment. The ankle will be passively moved at a velocity of 0.4°/s in plantar flexion, dorsal flexion, inversion, and eversion direction by the platform. Once the participant could detect the motion of the leg, they will be asked to press a stop button to stop the platform. The participants will be asked to confirm the direction of the motion in case they are guessing the movements. The angular movement of the platform will be recorded as the threshold for the detection of the ankle joint movement. Three trials will be performed for each direction on each foot. The mean of three trials will be used for the analysis.

## Dynamic leap and balance test (DLBT)

DLBT is a novel test that involves a dynamic task with serial changes in the base of a sport, such as walking and running, to assess balance [23]. The pattern of the DLBT will consist of 11 total targets (one central target and two peripheral targets along each of the five directions) as the medial half of the SEBT for each foot. The short and long targets will be placed at 100% and 150% of the distance based on the average normalized SEBT reach distances. Each leap will require the participant to leap with one leg and land on the other. The time that the participant leaps from the central target to the peripheral target and back to the central target in the order of 1–10 will be recorded. The faster the participants perform, the better their condition will enhance. They will be asked to stabilize for 2 s each time they returned to the central target before jumping to the peripheral target. The mean values of the three trials will be used for the analysis.

### Participant timeline {13}

The time schedule of enrolment, visits for participants, intervention, and assessments are shown in Fig. 3.

### Sample size {14}

The sample size is estimated on G* Power software (Germany) on the basis of a previous study [12], which reported that the CAIT score increased from 17.6 ± 2.1 cm to 28.4 ± 2.4 cm after 12 weeks of Tai Chi training. Under a significance level of 0.05, a statistical power of 0.80 in the two-tailed test, and a drop-out rate of 20% [24], the effect size and estimated required sample size of two groups are 0.57 [12] and 104, respectively.

### Recruitment {15}

Recruitment will be carried out at Shandong Sport University in Shandong, China. Leaflets, posters, and recruitment advertisements will be distributed on WeChat. All potential participants will be screened by the research assistant with professional qualification for participation based on the inclusion and exclusion criteria listed above. After being initially screened for inclusion and exclusion criteria, eligible volunteers who agree to participate will sign a written informed consent before the intervention. Through the informed consent, the purpose, procedures, potential risks, benefits, and confidentiality of the information will be explained in detail. The informed consent process will be performed by the main researchers (HT). On the consent form, participants have the right to withdraw at any time and will be asked if they agree to the use of their data should they choose to withdraw from the trial. They will also be asked for permission for the research team to share relevant data with people from the universities taking part in the research or from regulatory authorities where relevant. This trial does not involve collecting biological specimens for storage. During the trial, the participants will be informed to avoid participating in any other regular rehabilitation training and maintain their previous lifestyle. There is no anticipated harm and compensation for trial participation. All subjects will be instructed to complete a questionnaire about the basic personal information and the medical history. They are available from the corresponding author on request. The patients were initially recruited on September 22, 2021.

## Assignment of interventions: allocation

### Sequence generation {16a}

After the baseline assessment, the participants will be randomly assigned to the STCEP group and the control group at a ratio of 1:1. SPSS version 22.0 (IBM, NY, USA) will be used for random allocation. A randomization sequence will be created by an independent research assistant.

### Concealment mechanism {16b}

Randomization will be placed into sealed opaque envelopes by an independent research assistant who is blinded about the trial and released later. Each participant’s identity will be represented by a serial number. The letters “A” and “B” represent the groups assigned, such as “STCEP group” and “control group.”

### Implementation {16c}

A randomization sequence will be created by an independent research assistant (A). The investigator (YC) will take charge of the participant recruitment (informing them the trial procedures and obtaining their informed consent before inclusion in the study). Furthermore, the research assistant (B) will assign the participants to interventions.

## Assignment of interventions: blinding

### Who will be blinded {17a}

Outcome assessors, data managers, and statistical analysts will be blinded to group assignment and letter representation. In addition, participants will be asked not to discuss their experiences during training if they incidentally encounter other participants.

### Procedure for unblinding if needed {17b}

Unblinding is permissible only when a serious adverse event or emergency rescue occurs.

## Data collection and management

### Plans for assessment and collection of outcomes {18a}

Two principal assessors will conduct a comprehensive training in the measures and be responsible for data collection. Interrater reliability will be determined, and discrepancies will be resolved by consensus. The questionnaires and laboratory tests used in this study have been widely used in other studies. They are available from the corresponding author upon request. Primary and secondary outcomes will be measured at baseline, 4th, 8th, and 12th weeks of training.

### Plans to promote participant retention and complete follow-up {18b}

Participants have the right to withdraw at any time and will be asked if they agree to the use of their data should they choose to withdraw from the trial. They will also be asked for permission for the research team to share relevant data with people from the universities taking part in the research or from regulatory authorities where relevant.

### Data management {19}

Some outcome data will be collected via the paper materials (writing participant identity in serial number). Data integrity and validity will be verified and independently entered by a research staff, and a duplicate copy will be stored in a separate password-protected hard drive. All data will be unmodifiable once input and checked. All computers and electronic systems will be kept in laboratories. Entry into laboratories was controlled by restricting all unauthorized individuals. Only the statisticians will have access to the database to conduct final statistical analyses.

### Confidentiality {27}

The information collected will be used for research purposes and analyzed under the protection of personal privacy. All personal information will not be shared or released.

### Plans for collection, laboratory evaluation, and storage of biological specimens for genetic or molecular analysis in this trial/future use {33}

Not applicable. This trial does not involve collecting biological specimens for storage.

## Statistical methods

### Statistical methods for primary and secondary outcomes {20a}

Data will be analyzed with SPSS 20.0 (IBMS, NY, USA) and expressed as mean ± standard deviation. Two-way repeated ANOVA will be used to determine the main effects of groups, intervention time, and interaction on the measurements. If any significant main and interaction effects will be found, the Bonferroni method will be conducted for post hoc comparisons. The significant level will be set at 0.05.

### Interim analyses {21b}

Not applicable. Interim testing will not be conducted in this study.

### Methods for additional analyses (e.g., subgroup analyses) {20b}

Not applicable. No subgroup will be established in this study.

### Methods in analysis to handle protocol non-adherence and any statistical methods to handle missing data {20c}

If any participant withdraws from the trial, the last observation-carried forward method and multiple imputation method will be respectively conducted to adjust for the missing data.

### Plans to give access to the full protocol, participant-level data, and statistical code {31c}

The datasets analyzed during the current study are available from the corresponding author upon reasonable request.

## Oversight and monitoring

### Composition of the coordinating center and trial steering committee {5d}

The group of the coordinating center will be composed of teachers in the College of Sports and Health, Shandong Sport University. The trial steering committee will consist of three main investigators in this study, the administrative staff of funding and process supervision.

### Composition of the data monitoring committee, its role, and reporting structure {21a}

Making plans to consider a data monitoring committee is unnecessary because this intervention is a low risk.

### Adverse event reporting and harms {22}

The trial will not use investigational medicinal products, and the researchers have already obtained the practicing certificate of a rehabilitation therapist with abundant clinical experience. The participant will be monitored and documented during the study intervention. Any adverse events (defined as any functional impairment caused by the intervention, such as ankle sprain, ankle pain, and pallor) will be recorded. If serious adverse events occur, the participants will immediately discontinue the intervention and receive appropriate treatments. Incidents will be reported to the Ethics Committee of Shandong Sport University.

### Frequency and plans for auditing trial conduct {23}

The Project Management Group will report the study progress in the form of weekly research meetings. The trial steering committee and ethics committee will regularly supervise the procedure of the trial every 4 weeks and make recommendations regarding the necessary protocol modifications of all or part of the study. In this trial, on-site monitoring will be adopted to review the trial processes.

### Plans for communicating important protocol amendments to relevant parties (e.g., trial participants, ethical committees) {25}

The participants, trial registry, and ethics committee will be informed of any protocol modifications by phone, including the principal investigator, informed consent form, study protocol regarding eligibility criteria, outcomes, and analyses.

## Dissemination plans {31a}

The findings of this study will be reported in a master’s thesis by the main author and submitted to a peer-reviewed journal for publication. It will be presented at relevant conferences on the subject of the matter if possible.

## Discussion

This study will focus on the effect of STCRP on the neuromuscular function of individuals with FAI. Examining neuromuscular activities during the performance of a dynamic task can provide further insights into neuromuscular strategies utilized by individuals with FAI to maintain postural stability.

Tai Chi movements are performed with frequent body weight shifts between both legs and characterized by a low impact and a low loading rate [24]. Therefore, the activation of the muscles around the ankle may be increased during practicing. Tai Chi is performed with ankle flexion and extension in various directions, which may increase stimulations to the proprioceptors of the ankle joint. Wang et al. reported that adequate and appropriate mechanical loading is necessary to maintain physiological joint homeostasis [24].

We propose that Tai Chi exercise can be an effective exercise intervention as an option for individuals with FAI. However, the biomechanical mechanism and efficacy of Tai Chi in the treatment of FAI remain unclear. In this experiment, a rigorous random parallel control design will be conducted to observe the effectiveness and investigate the biomechanical mechanism of Tai Chi training on patients with FAI. This trial will provide new data and insights into the effectiveness of TC exercise and its influence on the clinical and functional aspects of FAI.

However, few limitations exist in this study. First, our sample size is small. Our recruitment strategy for this study (only in school) may have introduced some selection bias. Second, the participants and the instructors in this trial cannot be blinded. Their subjective expectations may skew the results.

In conclusion, this study attempts to estimate the effect of Tai Chi intervention on the neuromuscular function of individuals with FAI. It may provide evidence to support the beneficial effects of a Tai Chi exercise program on the neuromuscular function of individuals with FAI. The findings of this study will fill the research gap in the efficacy of Tai Chi based on the results of the proposed project. Further comprehensive research on the exercise rehabilitation of FAI will be proposed. Furthermore, the possible mechanism of Tai Chi on the neuromuscular function of patients with FAI may be discussed.

## Trial status

The protocol is in version 1.0. The patients were initially recruited on September 22, 2021, and the trial is currently underway and expected to be completed by July 1, 2022.

## Abbreviations

CAI: Chronic ankle instability
CAIT: Cumberland ankle instability tool
DLBT: Dynamic leap and balance test
sEMG: Surface electromyography
FAI: Functional ankle instability
mSEBT: Modified Star Excursion Balance Test
STCEP: Simplified Tai Chi exercise program
